# Microbial involvement in Alzheimer disease development and progression

**DOI:** 10.1186/s13024-020-00378-4

**Published:** 2020-07-24

**Authors:** Hannah R. Bulgart, Evan W. Neczypor, Loren E. Wold, Amy R. Mackos

**Affiliations:** 1 Biomedical Sciences Graduate Program, College of Medicine, The Ohio State University, Columbus, OH USA; 2 Dorothy M. Davis Heart and Lung Research Institute, College of Medicine, The Ohio State University, Columbus, OH USA; 3 College of Nursing, The Ohio State University, 1585 Neil Ave, Columbus, OH 43210 USA; 4 Department of Physiology and Cell Biology, The Ohio State University College of Medicine and Wexner Medical Center, Columbus, OH USA

**Keywords:** Gut microbiota, Oral microbiota, Alzheimer disease

## Abstract

Alzheimer disease (AD) is the most prominent form of dementia and the 5th leading cause of death in individuals over 65. AD is a complex disease stemming from genetic, environmental, and lifestyle factors. It is known that AD patients have increased levels of senile plaques, neurofibrillary tangles, and neuroinflammation; however, the mechanism(s) by which the plaques, tangles, and neuroinflammation manifest remain elusive. A recent hypothesis has emerged that resident bacterial populations contribute to the development and progression of AD by contributing to neuroinflammation, senile plaque formation, and potentially neurofibrillary tangle accumulation (Fig. 1). This review will highlight recent studies involved in elucidating microbial involvement in AD development and progression.

## Background

Alzheimer Disease (AD) is a progressive neurodegenerative disease characterized by short- and long-term memory loss, impaired decision making, forgetfulness, and changes in mood. Disease progression varies considerably across the population and can be classified as familial AD or sporadic AD. Familial AD often leads to early-onset AD, characterized by symptom progression before the age of sixty-five, and is linked to gene mutations in the amyloid precursor protein (APP), presenilin-1 (PSEN1), and presenilin-2 (PSEN2) genes [1]. Conversely, sporadic AD, occurring in 90% of cases and typically involving disease onset after age 65, is a multivariable process determined by a wide variety of genetic and environmental factors. Regardless of the timing of disease onset, the two hallmark features of both familial and sporadic AD are aberrant accumulation of extracellular amyloid-beta (Aβ) in senile plaques and intracellular neurofibrillary tangles (NFT) of hyperphosphorylated tau [2]. While it is known that Aβ plaques and NFT contribute heavily to neuronal death, it is unclear as to why these proteins accumulate in the first place. There is gaining evidence suggesting that the development of many of the hallmark features of AD, including Aβ plaques and NFT, can be linked to microbes that naturally reside in the body. This review will highlight the potential implications of resident microbes on the development and progression of AD.

### Senile plaques and neurofibrillary tangles

The most notable theory of AD development involves heightened accumulation of amyloid fragments due to altered APP processing, a transmembrane protein that is normally responsible for synaptic stability, neuronal protection, and neuronal growth [3–5]. APP has been known to have a positive effect on brain tissue via regulation of neurogenesis and neuronal proliferation [6–8], but when cleaved improperly it can give rise to Aβ which, in turn, can accumulate in excess and facilitate neuronal death [4]. Normal APP processing is achieved by an initial cleavage of APP by α-secretase, followed by γ-secretase, leading to soluble APPα (sAPPα). Duplications of the APP gene, as a result of a gene duplication event or duplication of the entire chromosome 21 as seen in Down syndrome, can result in the upregulation of APP which can be processed into Aβ and cause increased plaque buildup and consequent downstream tau phosphorylation and NFT pathology [1]. Other known APP mutations alter the conformation of α, β, and γ secretase cleavage sites resulting in altered peptide lengths and increased Aβ accumulation [1, 9]. PSEN1 and PSEN2 are γ secretase subunits that are normally involved in memory, neuron survival, and synaptic function [10]. Mutations in PSEN1/2 lead to abnormal C-terminal cleavage of APP leading to two Aβ isoforms: Aβ_40_ and Aβ_42_ [1]. Of the two isoforms, Aβ_42_ is more toxic and prone to accumulation than its shorter relative, Aβ_40_ [11]. Potentially pathogenic APP processing, i.e. that gives rise to Aβ, occurs when APP is initially cleaved by beta-site APP-cleaving enzyme 1 (BACE1) then by γ-secretase, leading to sAPPβ [4, 12]. Following APP processing, Aβ is released extracellularly via exocytosis where it can accumulate to form soluble oligomers [4]. Small amounts of soluble Aβ are normally produced in the brain; however, it is normally cleared through mechanisms including proteasomal, lysosomal, and/or enzymatic degradation [13]. In an AD brain there is insufficient degradation of Aβ, leading to an accumulation of Aβ fragments into senile plaques [14]. Soluble Aβ has been associated with decreased synaptic activity and weak nerve impulses, which suggests that Aβ contributes to synapse injury [15].

Tau is an essential neuronal microtubule-associated protein that tends to localize to the axon due to its main function of stabilizing the formation of microtubules to provide structural support for the neuron [16]. There are several cellular processes in the brain that are dependent on tau, such as axonal growth and vesicle/organelle transport [2]. The phosphorylation of tau at Ser214 and Thr231 can lead to the disruption of these cellular processes due to the detachment of tau from the microtubule [2, 17]. In a normal brain there is a balance of phosphorylated and non-phosphorylated tau that is regulated by the balance of activity between tau kinases and phosphatases [2]. In an AD patient, the tau kinase/phosphatase balance is impaired, with the balance being skewed towards tau kinases leading to hyperphosphorylated tau [2]. When tau is hyperphosphorylated, it assembles into tangles [3]. When the hyperphosphorylated form of tau is prominent, cellular processes that rely on tau are disrupted and axonal and dendritic transport are compromised. Interestingly, Aβ contributes to phosphorylated tau protein via activation of tau kinases [3].

### Amyloid as an antimicrobial peptide

The highly conserved nature of Aβ accumulation has been proposed to have a beneficial function [18], whereby it can function as an antimicrobial peptide (AMP) due to numerous shared characteristics including β-sheet structures, the ability to oligomerize and fibrillize, and the ability to destroy microbes (Table 1) [19, 20, 39]. In 2010, Soscia et al. compared the antimicrobial activity of Aβ and a widely accepted AMP, LL-37, against twelve microorganisms including *Esherichia coli*, *Streptococcus pneumonia*, *Streptococcus salivarius,* and *Candida albicans* [20]. Results showed that Aβ was able to inhibit the growth of all twelve microorganisms tested and in some cases Aβ was more potent than LL-37 [20]. In 2016, Kumar et al. demonstrated that Aβ accumulation was protective against bacterial-induced murine encephalomyelitis in an APP/PSEN1 transgenic mouse compared to non-transgenic littermates [19]. In this study, it was also demonstrated that higher mortality rates were observed in mice following bacterial inoculation of the brain in which a lower amount of Aβ was expressed. This result was attributed to the overgrowth of pathogen in the brain [19]. Bacteria, including oral pathogens and various spirochetes, are commonly found associated with Aβ plaques (Table 1) [21, 22, 40] . Aβ peptides show protective capabilities when the body, specificity the brain, encounters pathogens, however the over accumulation of Aβ peptides, either due to perpetual pathogen colonization or the inability to clear Aβ once it is no longer needed, can lead to destruction of nearby tissue due to plaque formation and hyperphosphorylation of tau [14, 19].

**Table 1:** Bacterial involvement in AD

Finding	Supported by	Model
Amyloid as AMP	19, 20	5XFAD mouse [19], in vitro [19, 20], nematode [19], human brain tissue [20]
Bacteria colocalizes with Aβ plaques	21, 40	Sprague-Dawley rats [21], Human brain tissue [40], specific pathogen-free BALB/c mice [40]
Ab fibrils activate microglia	45	Human THP-1 monocytes and microglia
EP2 induces neuronal damage by toxicity and increased amyloid beta levels	49	APPSWE-PS1△E9 mice
Overactive microglia lead to neuroinflammation	53	APP/PS1 mice
LPS is more abundant in AD brain	99, 100	Human brain tissue
LPS stimulation leads to enhanced Aβ accumulation	23, 24	*E. coli* cultures [23], APP Swe Tg mice [24]
AD induces changes in bacterial communities	25-29	APP/PS1 mouse stool [25], human stool [26–28], human brain tissue [29]
Broad-spectrum antibiotic cocktail altered gut bacterial communities and reduced AD hallmark characteristics	89	APPSWE/PS1ΔE9 mice
Rifampicin treatment reduced AD hallmark characteristics	30-33	Cell culture [30–32], APP_OSK_ mice [32, 33]
Minocycline treatment reduced AD hallmark characteristics	34, 35	Sprague-Dawley rats [34], APP Tg mice [35]
Periodontal disease risk factor for AD	112	Human patient serum
*P. gingivalis* can access brain and associate with Aβ plaques	36-38, 40	Human brain tissue [36, 40], ApoE^−/−^ mice [37, 38], specific pathogen-free BALB/c mice [40]
AD patients have increased antibodies to periodontal disease-associated microbes	112, 114	Human patient serum
Probiotic supplementation improves cognitive function and reduces neuroinflammation	102, 103	Human

### Neuroinflammation

Neuroinflammation has been tightly linked to AD pathogenesis. It has been proposed that neuroinflammation exacerbates hallmark AD characteristics including Aβ deposits and tau hyperphosphorylation leading to tissue damage, which can further the inflammatory response, creating a vicious cycle of inflammation and tissue destruction [41, 42]. Pro-inflammatory cytokines associated with AD are interleukins (IL)-1β, IL-6, IL-12, IL-18, tumor necrosis factor (TNF)-α, TNF-β and interferon (INF)-γ [41]. In the AD brain, the concentrations of IL-1β, IL-6, IL-12, IL-18, and TNF-α are significantly greater than a non-AD brain [41]. The purpose of these cytokines is to protect the tissue from pathogens, however host tissue is also susceptible to the destructive nature of the inflammatory response, thus uncontrolled or excessive inflammation can enhance tissue damage and contribute to AD pathogenesis [41].

The two main cell types involved in neuroinflammation are microglial cells and astrocytes. Microglial cells are part of the innate immune system and function to maintain neuronal homeostasis by removing dead/dying cells, cellular waste, and Aβ without the induction of the inflammatory response [43, 44]. Additionally, microglia act as a surveillance service to detect pathogens and/or tissue damage. Numerous substances including pathogen-associated molecular patterns (i.e. lipopolysaccharide and peptidoglycan), damage-associated molecular patterns, and Aβ fibrils activate microglial cells (Table 1) [45]. Once activated, the microglial cell can produce proinflammatory cytokines and free radicals in an effort to protect the tissue against the pathogenic insult [43, 46, 47]. Microglial activation is highly associated with neurotoxicity and inflammation, which can further damage the tissue (Table 1) [48, 49]. Prostanoid subtype 2 receptor (EP2), the receptor for prostaglandin E2, has been associated with the toxic effects of microglial activation [48, 49]. When EP2 is knocked out, neuronal damage due to neurotoxicity is reduced, phagocytosis of Aβ is increased, and Aβ levels are reduced [48, 49]. Furthermore, when microglia become over activated, they lose their ability to effectively phagocytose Aβ and begin to release pro-inflammatory cytokines, which also leads to neuroinflammation [50–53]. Microglia appear to “age” and become dysfunctional throughout the human lifespan, especially in AD patients [50].

The other cell type involved in neuroinflammation is the astrocyte, which are normally involved in neurotransmission and preservation of the blood-brain barrier (BBB). Astrocytes are glial cells that are activated early in AD progression by increased levels of calcineurin and by Aβ accumulation [52, 54, 55]. Such activation causes astrocytes to stop clearing Aβ by use of apolipoprotein and to instead secrete Aβ and suppress the phagocytic function of microglia [52, 54, 55]. In mouse models of AD, blockage of the calcineurin signaling pathway responsible for astrocyte activation has been shown to decrease the Aβ plaque burden and improve cognitive and synaptic function [55]. Because it has been demonstrated that large quantities of Aβ42 accumulate in astrocytes, it is believed that astrocytes are heavily involved in Aβ deposition [56]. Evidence has revealed that astrocytes internalize Aβ in an effort to store it, which can lead to neuronal toxicity in the AD brain [57, 58].

## A nontraditional view of AD

Alzheimer Disease is traditionally considered a disease of the brain with little emphasis on other systems. However, evidence suggests that neural AD pathology is co-dependent with cardiovascular health and other physiological factors such as immune function and resident and/or pathogenic microbes. It is recognized that environmental and genetic factors each play a significant role in the development and progression of sporadic AD and include cerebral ischemia, elevated blood pressure, type 2 diabetes, low and high body weight, metabolic syndrome, cigarette smoking, and traumatic brain injury [59]. Due to recent evidence of the involvement of microbes in Aβ development, we propose the addition of a new risk factor: the microbiota. It is largely recognized that GI microbes can have a profound impact on systemic immunity, inflammation, barrier integrity, and neurological activity. Evidence suggesting the antimicrobial properties of Aβ has led to additional hypotheses linking microbial imbalances and microbial brain invasion to Aβ accumulation [60, 61]. Imbalances in the human microbiota as a result of diet, chronic infection, environmental exposures, or the aging process could lead to a physiological response of increased Aβ production and accumulation, which could contribute to Aβ saturation prior to AD symptoms and widespread NFT pathology [60, 62].

### Microbiota

The human body is colonized by a vast array of microbial organisms, collectively known as the microbiota. Proportionally, the human microbiota consists mostly of bacteria, however fungi, viruses, archaea, and protozoa are also present to a lesser degree, thus herein when referring to the microbiota we will refer to bacterial populations [63]. New estimates have calculated that there are nearly 4 × 10^13^ bacterial cells in the average 70 kg male human body, which is roughly a 1:1 ratio with the number of human cells present [64]. Bacterial colonization begins at birth which is critical in early life because microbes assist in the development of the gastrointestinal (GI) tract, the brain, and train our immune system [65]. Microbes also provide nutrients and energy for the host through fermentation, degrade potentially toxic substances, and play a key role in the protection of the body against pathogen colonization [65, 66]. Microbes colonize nearly all surfaces that are exposed to the outside environment, including the skin, respiratory tract, and oral cavity, but the vast majority of bacteria in the body are found in the GI tract with the most diverse and dense population residing in the distal colon [63]. The microbiota also aides in controlling proliferation and differentiation of epithelial cells in the gut through the production of short chain fatty acids, such as butyrate [66]. It is important to note that gut bacteria play a significant role in the GI tract, but also throughout the entire body [67].

The human microbiota is linked to several beneficial physiological effects, however some resident microbes are opportunistic pathogens. Host organisms are considered pathogenic if they disrupt the immune system or produce harmful metabolites or enzymes [68]. Many harmful bacterial metabolites produced are associated with, but not limited to cardiovascular disease, kidney disease, several cancers, and neurodevelopment disorders [68]. While fluctuations in the microbiota can elicit physiologic dysfunction, the resident microbes are essential to host physiology and function, thus the beneficial effects greatly outnumber the negative effects.

The structure and function of the bacterial community is relatively stable and is largely dependent on the site of colonization. The community structure is sensitive to host and environmental factors including changes in host diet, antibiotic use, psychosocial stressor exposure, and host immune factors [69, 70]. Upon exposure to these factors, there can be changes in the relative abundance of bacterial species that can lead to an imbalance in the composition and function of the resident microbiota, termed dysbiosis. Generally these shifts in the community structure are short lived and once the stimulus is removed, the resident populations will come back to steady state; however, if the stimulus cannot be removed, such as in individuals with inflammatory diseases, the dysbiosis can continue and potentially contribute to disease. This is important because bacteria, particularly gut bacteria, have been associated with many diseases including cardiovascular disease, inflammatory bowel disease, obesity, diabetes, some cancers, and numerous neurological disorders [71].

### Gut-brain-microbiota Axis

The largest and most genetically diverse bacterial community in the body resides in the GI tract where populations range from 10^4^ to 10^7^ organisms per gram of contents in the stomach and duodenum to 10^11^ organisms per gram of contents in the distal colon [72]. It is estimated that upwards of 50 bacterial phyla and nearly 1000 bacterial species inhabit the healthy gut. These resident microbes have been shown to modulate the activities of distant sites including the brain via the bidirectional communication of the GI tract through interactions between the enteric nervous system (ENS) and central nervous system (CNS) in what is known as the gut-brain-microbiota axis [73]. It has been widely established that the vagus nerve, which extends from the brainstem to the esophagus, stomach, and intestines, is responsible for relaying impulses from the brain to the GI tract and vice versa with the majority of the communication occurring from the GI tract to the brain. Vagal fibers do not penetrate the GI epithelium and therefore do not directly contact the gut microbiota under normal conditions [74]. Even without direct contact, gut microbes can interact with the ENS and the vagus nerve indirectly via the production of neurotransmitters, short chain fatty acids, or bacterial metabolites. Indirect interactions can also be mediated by enteroendocrine cells, which can detect signals from the microbiota and relay them to the vagal fibers [75]. Studies have also suggested that bacteria-host communications can be mediated by host hormones and hormone-like bacterial autoinducers [76, 77]. Gut-brain-microbiota interactions can have a wide array of effects on GI and CNS properties, including pain sensation [78, 79], various digestive properties [80, 81], response to stress [82], cognitive function [83] and possibly emotions [84].

Gut microbes play a significant role in educating the immune system, so it is possible that dysbiosis can lead to improper immune activation. Overactive microglia and enhanced inflammation are both indicated in behavioral disorders and numerous neuronal diseases, including AD. Gut microbes have been linked to both BBB permeability and microglial maturation and function [85]. Germ-free studies have also demonstrated that mice devoid of microorganisms have abnormal brain development, experience learning and memory deficits, and have enhanced anxiety-like behavior [86, 87]. Often such physiologic traits can be diminished by the recolonization of gut microbes from conventional mice [87].

### Gut microbiota and AD

The assessment of resident microbes in the development and progression of AD is still in its infancy. Genetically modified mice in which Aβ plaque formation is enhanced or tau phosphorylation is accelerated have been a valuable tool to determine host-microbe interactions in the context of AD. In vitro studies have also demonstrated that bacterial components such as lipopolysaccharide (LPS), a highly immunogenic cell wall component of Gram negative bacteria, and bacterial DNA can lead to enhanced Aβ accumulation and tau misfolding (Table 1) [23, 88]. This was further demonstrated in vivo by Sheng et al. via intraperitoneal administration to of *E. coli*-derived LPS to APPswe mice, which lead to enhanced neuroinflammation and neuronal Aβ accumulation (Table 1) [24]. In APPPS1mice there are significant changes to the gut bacterial community structure as compared to aged-matched wild type mice. Differences include significant reductions of phylum members of Firmicutes, Verrucomicrobia, Proteobacteria, and Actinobacteria and significant increases in Bacteroidetes and Tenericutes in APPPS1 over that of non-transgenic mice (Table 1) [25]. Interestingly, when APPPS1 mice were raised in germ-free conditions, i.e. devoid of all microbes, neuronal Aβ accumulation was significantly reduced as compared to conventionally-raised transgenic mice, possibly due to an increase in enzymes involved in degrading Aβ [25]. It is important to note that Aβ plaque development was not fully blocked in the absence of the microbiota.

Antibiotic treatment and germ-free studies have demonstrated the involvement and importance of gut microbes in AD. Antibiotic treatment in AD mice has been associated with reduced fibril formation, reduced Aβ toxicity, and increased memory and learning [30–33]. Two antibiotics that have been utilized in numerous animal and human studies are rifampicin and minocycline. Rifampicin is a broad-spectrum antibiotic that can cross the BBB. In vitro studies have demonstrated that rifampicin prevented the production of Aβ aggregates and protected from Aβ-associated cellular toxicity (Table 1) [30, 31]. Additionally, Umeda *et. al* demonstrated that rifampicin reduced the amount of Aβ deposits, tau phosphorylation, microglial activation, and improved the memory in APP_OSK_ mice (Table 1) [32, 33]. Similar to rifampicin, minocycline can also cross the BBB and has anti-bacterial properties along with anti-inflammatory and proteolysis inhibition properties. Several studies have demonstrated that minocycline administration reduces neuronal Aβ levels, tau phosphorylation, and neuroinflammation (Table 1) [34, 35]. Although minocycline has been shown to reduce Aβ, Cai et al. found that APP levels were unaffected by antibiotic treatment leading the investigators to hypothesize that minocycline-dependent reductions of neuroinflammation (i.e. IL-1β and TNF-α) lead to the reduced Aβ [34]. Unfortunately in the aforementioned rifampicin and minocycline studies, resident bacterial populations were not assessed. A recent study conducted by Minter et al., demonstrated that long-term treatment of APP_SWE_/PS1_ΔE9_ mice with an antibiotic cocktail throughout post-natal development leads to reduced Aβ plaque accumulation and size and reduced gliosis in the vicinity of Aβ plaques [89]. Furthermore, there were reductions in circulating proinflammatory cytokines and chemokines, which was correlated to significant alterations of alpha and beta diversity in antibiotic-treated mice [89]. This study effectively links microbial reprogramming to altered host immunity and AD pathogenesis, but the mechanisms linking these phenomena remain unclear.

Human studies assessing the gut microbial composition in AD patients have demonstrated that gut microbes from AD patients experience reduced alpha diversity and distinct shifts in beta diversity [26–28]. An association between irritable bowel syndrome and risk of dementia has been established, and gut dysbiosis is hypothesized to be a factor in this risk association [90]. Additionally, a novel computational algorithm analyzed existing GI microbial metabolite data and found 56 metabolites to be associated with cognitive decline [91]. Similar to animal studies, AD patients reportedly experience phyla level alterations, but currently there is not a consensus on which phyla are more abundant or less abundant. Vogt et al. showed reductions in Firmicutes and Actinobacteria and an increased abundance of Bacteroidetes, all of which support findings in the APPPS1 mouse model [26]. Liu et al. corroborated the reduction in Firmicutes, and also found an increase in Proteobacteria [27]. Alternatively, Zhuang et al. showed a slight, yet significant, decrease in Bacteroidetes, and an increase in Actinobacteria in AD patients, both of which contradict Vogt et al., Liu et al., and the APPPS1 mouse model [28]. Zhuang et al. also found an increase in the Firmicutes/Bacteroidetes ratio in AD patients, while Vogt et al. and Liu et al. found a decrease. This ratio has consistently been shown to increase alongside body mass index (BMI) [92]. Vogt et al. and Liu et al. reported similar BMI across their groups, however BMI was not reported in the Zhuang et al. study and could have affected their reported phyla alterations if BMI was not properly controlled [26–28]. The relationship between AD development and obesity is complex, as increased mid-life BMI is an established risk factor for AD [93], but increased late-life BMI has been proposed to be protective against AD [94]. However, the detection of this apparent late-life protection may be confounded by proactive weight loss during preclinical AD, i.e. patients at risk of AD losing weight in hopes of reducing their disease severity [92].

Each of these human studies utilized similar exclusion and diagnostic criteria, used age/sex-matched controls, and controlled for antibiotic use prior to sample collection. The three study sites differed: Wisconsin, USA [26], Hangzhou, China [27], and Chongqing, China [28], and the studies did not control for diet, a large contributor to the microbial community structure. Regional differences have been shown to impact phyla compositions in healthy subjects, with Chinese individuals having Bacteroidetes-rich and Firmicutes-poor guts compared to those in the US [95]. Baseline differences in microbial community structure could reasonably affect potential AD-associated alterations. Further, collection methods were different in that researchers either collected stool from each patient [27, 28] or relied on self-collected samples by patients in their homes and delivered to the researchers the following day [26]. The differences in study locations and methodologies could partially account for the conflicting results, but each study had small experimental and control group sizes of 25 [26], 33 [27], and 43 [28]. These inconsistent results may be attributed to poor study power leading to false positives [96]; therefore, interpreting AD-associated gut phyla alterations should be done with caution until a strong consensus is established through high-powered multi-regional studies. To build on these studies of gut phyla, Emery et al. found that Actinobacteria was the most abundant bacterial phylum in postmortem AD brain samples, however this low powered pilot study requires corroborating evidence before making interpretations and generalizations [29].

Overall it is still unclear if/how the phyla are altered due to conflicting studies. It is possible that assessing bacterial phyla/genera variations in AD may not be as useful as quantifying AD-associated alterations of microbial metabolites. A recent study demonstrated that an altered gut microbiota lead to increased levels of circulating phenyalanine and isoleucine, which lead to increased neuroinflammation in the 5XFAD mouse model [97]. The increased phenylalanine and isoleucine were also demonstrated in two separate cohorts of AD patients. This lead to a treatment, i.e. GV-971, a sodium oligomannate, which stabilizes the gut microbiota, reduces circulating phenylalanine/isoleucine, and ameliorates cognitive impairment in AD patients [97].

Studies of human stool samples have found that patients with cognitive impairment and brain amyloidosis experience increased levels of potentially noxious microbes, namely *Escherichia* and *Shigella*, and reductions in protective microbes including *Eubacterium rectale* in comparison to amyloidosis-free controls with and without cognitive impairment [98]. These alterations are accompanied by increased inflammatory markers in the circulation, thus raising the possibility that gut dysbiosis may lead to systemic inflammation, resulting in amyloidosis and AD progression. Importantly, this study was correlational in nature and experimental models are needed to convincingly connect these findings. LPS has also been shown to be more abundant in the AD brain and is found to be associated with Aβ plaques (Table 1) [99, 100]. It is possible that the increased abundance of LPS-producing bacteria can activate immune cells leading to enhanced inflammation that is observed in AD patients. In addition to its effects on neurons and immune cells, addition of LPS to human blood samples has been shown to cause quicker formation of firmer clots [101]. Notably, AD patients are known to have hypercoagulable blood which can contribute to neuroinflammation and cognitive decline [101].

The use of probiotics, i.e. microbes with a known beneficial effect on health, to prevent or remedy dysbiosis in an effort to treat or prevent dysbiosis-induced disease is an area of increased investigation. Few studies have investigated the use of multispecies probiotic cocktails in AD patients. In a randomized, double-blinded, controlled clinical trial of 60 AD patients, the authors demonstrated that 12 weeks of probiotic supplementation significantly improved the Mini-Mental State Examination scores as compared to non-probiotic treated subjects (Table 1) [102]. Unfortunately, gut microbes were not assessed in this trial. An additional study was conducted in an effort to stabilize the gut microbiota in an attempt to reduce systemic and neuroinflammation via probiotic intervention (Table 1) [103]. In this study, 20 AD patients were supplemented with a multispecies probiotic cocktail for one month. Results demonstrated a significant increase in serum kynurenine and increases in fecal zonulin and *Faecalibacterium prausnitzii*, however there was no change in cognitive function [103]. As this was just a pilot study, it is possible that the use of proper controls, a larger sample size, and more inclusive sequencing techniques could provide more insight into the use of probiotic supplementation in AD patients [104]. However, a greater issue is the use of probiotic supplementation as there is currently no consensus on which species/strain to use or how many colony forming units need to be consumed. Until we have a better understanding of the mechanisms by which probiotics exert their beneficial effects, we will likely see studies with vastly different results.

### Oral microbiota and AD

The GI tract begins in the oral cavity where a diverse array of microbes reside. The mouth harbors over 700 bacterial taxa, most of which reside in the anaerobic environment of the subgingival surface as a biofilm, more commonly referred to as dental plaque [105]. The physical removal of the subgingival biofilm, either through brushing the teeth or routine dental cleanings, is necessary to prevent periodontal disease, caries formation, and tooth loss. A consequence of this biofilm removal is that we experience bacteremia each time we brush our teeth or have a dental cleaning [106]. Generally, this bacteremia is transient, and bacteria are quickly cleared from the blood; however, in some individuals, oral microbes can leave the oral cavity and colonize distant sites in the body such as the heart (i.e, as with infective endocarditis), and possibly the brain. Immunosuppression and cardiovascular defects contribute to the likelihood of bacterial dissemination following dental procedures, however it is possible that oral dysbiosis can contribute as well [107, 108].

One major consequence of oral dysbiosis is the acquisition of pathogenic organisms or a bloom in opportunistic pathogens that can lead to periodontitis resulting in inflammation and pathogen-induced toxin formation. The resulting inflammation and toxin formation can lead to tissue destruction and even tooth loss, which in turn can increase the likelihood of bacterial dissemination. Oral microbes associated with periodontal disease include, but are not limited to, *Porphyromonas gingivalis, Treponema denticola, Tannerella forsythia,* and *Aggregatibacter actinomycetemcomitans*. Of these microbes, *P. ginigivalis* is considered a keystone periodontal pathogen in which its colonization alone can disrupt both host tissue and resident oral microbes [109, 110].

Non-AD individuals with periodontal disease have an increased amyloid load in the brain [111] and periodontal disease has recently been defined as a risk factor for AD development (Table 1) [112, 113]. The majority of the studies determining the link between AD and periodontal disease focus on *P. gingivalis*. As a member of the Bacteroidetes phylum, *P. gingivalis* is a Gram-negative bacterium that produces cysteine proteases known as gingipains that contribute to tissue destruction, allowing *P. gingivalis* and other pathogenic microorganisms to flourish. Another virulence factor of *P. gingivalis* is its ability to avoid immune detection through the suppression of adaptive immunity. It is hypothesized that the combination of immune system avoidance and gingipain-induced local tissue destruction allows *P. gingivalis* to escape from the mouth and migrate to the brain where it is thought to gain access and colonize through the use of gingipains. Both human and animal studies alike have demonstrated that not only can *P. gingivalis* gain access to the brain but that it also is associated with Aβ plaques (Table 1) [22, 36–38]. It has been hypothesized that, due to its antimicrobial properties, Aβ is produced as a mechanism to clear pathogens; however, as human brains can only be assessed post-mortem, the timing of bacterial colonization/Aβ accumulation is unknown. A recent study demonstrated that oral inoculation of BALB/c mice with *P. gingivalis* can lead to eventual brain colonization, subsequent Aβ plaque accumulation, and tau destruction [22]. Colonization, Aβ accumulation, tau tangles, neuroinflammation, and neuronal destruction were all linked to gingipain production, in that if a gingipain mutant of *P. gingivalis* or gingipain inhibitors were used, the effects were greatly diminished. The evidence that Aβ may accumulate in the brain in an effort to clear infectious organisms may explain why clinical trials aimed at the blockade of Aβ have had detrimental effects on patients. The new discovery of the effects of small-molecule inhibitors of ginigpains has led to ongoing clinical trials aimed at mediating *P. gingivalis* brain colonization and subsequent neurodegeneration that seems all too common in AD patients.

Longitudinal studies have found increases in serum antibodies to several periodontal disease-associated microbes including *Fusobacterium nucleatum* and *Prevotella intermedia* [112], as well as *Actinomyces naeslundii,* and *Eubacterium nodatum* [114] in AD patients versus age-matched controls years before the onset of AD symptoms. These two similar studies differed in the specific bacterial populations assessed, which prevented reproduction of results between two. Regardless, these observations support the hypothesis connecting oral dysbiosis to the development of AD. Additionally, spirochetes have been associated with AD brains because they have the ability to penetrate the CNS. Oral *Tremponema* is a gram-negative spirochete that is primarily associated with adult periodontitis [115]. In 2002, Riviere et al. observed significantly more *Treponema* species in AD brains than non-AD brains [116]. Using PCR analysis, they found a connection between AD and oral *Treponema.* The study suggests that oral bacteria can penetrate the central nervous system in AD patients and that spirochetes are incorporated in Aβ plaques. Recently Allen et al. confirmed that senile plaques form biofilms similarly to spirochetes [40]. The significance of this study was that senile plaques had similar characteristics as spirochetes in the AD brain.

### Mycobiota and AD

The fungal microbiota, referred to as the mycobiota, is estimated to represent ≤0.1% of the total human microbiota [117]. Similar to bacteria, fungi can be found on mucosal surfaces, and on the skin. The mycobiota has been largely understudied until recently due to the advancement in culturing techniques and next-generation sequencing. This advancement has led to the discovery of more resident fungal species, along with additional colonization sites within the body. How the mycobiota plays a role in health and disease is currently under intense study, and it has been associated with various diseases, one of which is AD.

There are currently few animal and human studies studying the effects of fungi on AD. At 3 months-post intravenous infection of *Candida glabrata* Pisa et al. were able to detect the fungal pathogen in the brains of nude mice (Table 2) [118]. Unfortunately, the development of Aβ plaques or additional AD neurologic biomarkers were not assessed. An additional study utilizing a murine model of low-grade *C. albicans* infection in the blood, i.e. candidemia, demonstrated bloodborne *C. albicans* can cross the BBB and lead to the development of Aβ plaques and increased activation of IL-1β, IL-6, and TNF-α (Table 2) [120]. Mice experiencing candidemia also experienced deficits in working spatial memory as early as 3 days post-challenge, which was reversible following the clearance of infection [120]. Lastly, it was determined that *C. albicans*-induced Aβ production aided in microglial phagocytosis and clearance of the pathogen, thus APP-deficient mice experienced impaired *C. albicans* clearance.

**Table 2:** Fungal involvement in AD

Finding	Supported by	Model
Fungal structures in AD brain colocalizes with Aβ plaques	118, 119	Human brain tissue [118, 119], nude mice [118]
Chitin structures in AD brain	121, 122	Human brain tissue
Fungi in CSF	123, 124	Human CSF
Aβ as an antifungal	20	Human brain tissue, in vitro
Bloodborne *Candida* can cross BBB	120	C57Bl/6, APP^−/−^, 5xFAD

Recent evidence has implicated fungal species with the etiology of AD. Human AD brain samples have demonstrated the presence of fungal cells which appear to colocalize with Aβ plaques, similar to observed findings with bacteria (Table 2) [118, 119]. Aβ has shown antimicrobial activity against fungus, particularly *C. albicans*, so it is plausible that plaque formation is occurring in response to a fungal infection [20]. Even though fungi have been shown in the AD brain in many studies, the size and cellular location vary from patient to patient. Pisa et al. reported fungal structures as small as 0.4 μm and as large as 10 μm [119]. At the cellular level, these fungal structures can be extracellular and intracellular, and intracellular structures can even be intranuclear [118, 119]. It was also noted that aberrant levels of cytoplasmic and nuclear tau was associated with fungi in human AD brain tissue, but this data has not been rigorously reproduced (Table 2) [119].

Within the AD brain, chitin polysaccharides, a key component of the fungi cell wall, have also been identified (Table 2) [121, 122]. Human cerebrospinal fluid (CSF) samples have also confirmed the presence of fungi in AD patients. Within the CSF of AD patients, fungal DNA, proteins, and associated molecules have been identified by multiple investigators (Table 2) [123, 124]. Alonso et al. identified fungi in all samples tested, however the sample size was small (*n* = 6) [123]. Species identified from AD CSF include *C. albicans*, *Cryptococcus*, *Malasezzia globosa,* and *Sacharomyces cerevisae* [123]. Interestingly, four out of six samples tested had multiple fungal species present in the CSF [123]. Currently, the role fungi play in the development and progression of AD has yet to be determined, but there is a growing amount of evidence that supports the presence of fungi in AD patients, which warrants further investigation.

## Conclusion

**Fig. 1:**
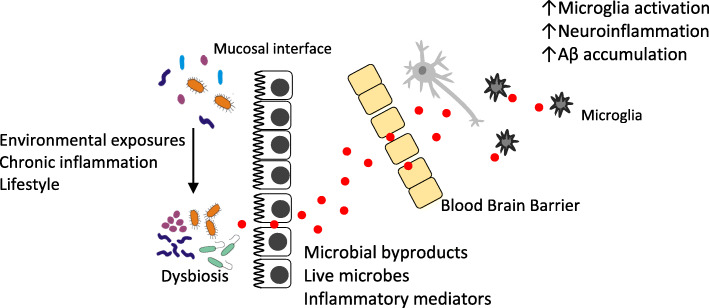
Potential mechanism by which distant microbial dysbiosis can affect AD development and progression

The mounting body of evidence has illuminated an intimate relationship between microbial dysbiosis and AD. New discoveries in this area are particularly exciting when considering the lack of an effective standard of care for AD patients seeking longer, higher quality lives. Although microbial imbalances are not the sole drivers of AD pathology, when investigating and treating diseases without a known cure, it is vitally important to understand all the contributing factors related to disease onset and progression. We present a potential mechanism by which environmental exposures or lifestyle factors contribute to the development of dysbiosis, which leads to increased mucosal inflammation, thereby loosening epithelial tight junctions allowing bacterial byproducts to enter the circulation (Fig. 1). The entry of these potentially noxious substances into the circulation may contribute to the development of a leaky BBB, thereby allowing their entry into the brain where they can induce neuroinflammation and Aβ accumulation which can contribute to the development of AD (Fig. 1). Further studies in animal models are needed to develop more in-depth mechanisms by which specific microbes and pathogens interact with the nervous and immune systems leading to diseased states. Inconsistent results in human studies of phlya alterations need to be reconciled through highly powered multi-region studies. Clinical and epidemiological studies focusing on preventative strategies, such as probiotic use and nutritional interventions, and potential treatments, such as microbial manipulation and fecal transplantation for AD are warranted. One challenge in drawing conclusions from these studies is the lack of a reliable diagnostic criterion for AD in live humans. As researchers continue to develop an understanding on this newfound relationship and investigate possible treatment strategies, clinicians should be advised to consider the evidence presented in this review outlining the potential impact of microbial imbalances on their patients’ AD progression.

## Data Availability

Not applicable.

## Abbreviations

Aβ: Amyloid beta
AD: Alzheimer disease
AMP: Antimicrobial peptide
APP: Amyloid precursor protein
BACE: beta-site APP-cleaving enzyme
BBB: Blood brain barrier
CNS: Central nervous system
CSF: Cerebrospinal fluid
ENS: Enteric nervous system
EP2: Prostanoid subtype 2 receptor
GI: Gastrointestinal
IL: Interleukin
INF: Interferon
LPS: Lipopolysaccharide
NFT: Neurofibrillary tangles
PSEN: Presenilin
sAPP: soluble amyloid precursor protein
TNF: Tumor necrosis factor
